# Fermentative Production of l-2-Hydroxyglutarate by Engineered *Corynebacterium glutamicum* via Pathway Extension of l-Lysine Biosynthesis

**DOI:** 10.3389/fbioe.2020.630476

**Published:** 2021-01-27

**Authors:** Carina Prell, Arthur Burgardt, Florian Meyer, Volker F. Wendisch

**Affiliations:** Genetics of Prokaryotes, Faculty of Biology, Center for Biotechnology (CeBiTec), Bielefeld University, Bielefeld, Germany

**Keywords:** *C. glutamicum*, L-2-hydroxyglutarate, metabolic engineering, glutarate hydroxylase, wheat sidestream concentrate, bioreactor

## Abstract

l-2-hydroxyglutarate (l-2HG) is a trifunctional building block and highly attractive for the chemical and pharmaceutical industries. The natural l-lysine biosynthesis pathway of the amino acid producer *Corynebacterium glutamicum* was extended for the fermentative production of l-2HG. Since l-2HG is not native to the metabolism of *C. glutamicum* metabolic engineering of a genome-streamlined l-lysine overproducing strain was required to enable the conversion of l-lysine to l-2HG in a six-step synthetic pathway. To this end, l-lysine decarboxylase was cascaded with two transamination reactions, two NAD(P)-dependent oxidation reactions and the terminal 2-oxoglutarate-dependent glutarate hydroxylase. Of three sources for glutarate hydroxylase the metalloenzyme CsiD from *Pseudomonas putida* supported l-2HG production to the highest titers. Genetic experiments suggested a role of succinate exporter SucE for export of l-2HG and improving expression of its gene by chromosomal exchange of its native promoter improved l-2HG production. The availability of Fe^2+^ as cofactor of CsiD was identified as a major bottleneck in the conversion of glutarate to l-2HG. As consequence of strain engineering and media adaptation product titers of 34 ± 0 mM were obtained in a microcultivation system. The glucose-based process was stable in 2 L bioreactor cultivations and a l-2HG titer of 3.5 g L^−1^ was obtained at the higher of two tested aeration levels. Production of l-2HG from a sidestream of the starch industry as renewable substrate was demonstrated. To the best of our knowledge, this study is the first description of fermentative production of l-2HG, a monomeric precursor used in electrochromic polyamides, to cross-link polyamides or to increase their biodegradability.

## Introduction

l-2-hydroxyglutarate (l-2HG) can be obtained by hydroxylation of the C_5_-dicarboxylic acid glutarate at the α-carbon position. Glutarate is known to be a demanded building block for the production of biopolymers, like aliphatic polyamide 6,5 which is mainly used in the construction industry (Navarro et al., 1997). Carbon chain length and functional groups of the monomers are important for the performance of the biopolymer with respect to physical and chemical properties. In the case of l-2HG, the additional hydroxyl group provides greater versatility for further chemical modification with the potential to alter the properties of the resulting polymer. Furthermore, incorporation of hydroxylated monomers enhances the biodegradability of the polyamide (Varela and Orgueira, 2000). Production of l-2HG by microbial fermentation would provide the chemical industry with an environmentally friendly building block for polyamides and polyesters. The addition of functionalities on polyester backbones (hydroxyl, carboxyl, allyl, azido, and acetylene groups) facilitates covalent post-polymerization modification. In 2017, it was shown that the enantiomer d-2-hydroxyglutarate (d-2HG) can be used as a functionalized building block for a polyester after cyclization to an allyl containing lactide (Nan and Feher, 2017). Also, it was demonstrated that electrochromic polyamides with functional hydroxyl groups for homogeneous hybrid films can be produced. The repeating units of hydroxysuccinate in the polymer backbone provided reaction sites for organic-inorganic bonding resulting in homogeneous and transparent hybrid films (Pan et al., 2018).

In medicine, l-2HG is used as a diagnostic biomarker for the characterization of various cancer types (Shim et al., 2014). Under oxygen limitation, l-2HG accumulates as metabolic intermediate in healthy as well as in malignant animal cells (Shim et al., 2014; Oldham et al., 2015; Shelar et al., 2018) facilitating a physiological adaption to hypoxic stress. The accumulation of l-2HG is triggered by increasing concentrations of 2-oxoglutarate (2-OG), which is caused by tricarboxylic acid cycle dysfunction and increased mitochondrial reducing potential. As consequence, the increased cellular l-2HG concentrations lead to inhibition of the electron transport and glycolysis compensating the effects of mitochondrial reductive stress induced by hypoxia (Oldham et al., 2015). The stereospecific reduction of 2-oxoglutarate to l-2HG is catalyzed by lactate dehydrogenase (LDH) and malate dehydrogenase (MDH) under hypoxic stress conditions (Intlekofer et al., 2017). In contrast, the formation of its enantiomer d-2-hydroxyglutarate (dr-2HG) is catalyzed by a mutated version of isocitrate dehydrogenase 1 or 2 (IDH1/2) contributing to the pathogenesis of cancer, whereas l-2HG biosynthesis does neither involve IDH1 nor IDH2. Albeit both enantiomers of 2-hydroxyglutarate display an inhibitory effect on 2-oxoglutarate-dependent enzymes involved in diverse biologic processes (Chowdhury et al., 2011; Xu et al., 2011), their stereospecific biosynthesis differs. Thus, bio-based routes enable stereospecific synthesis of either d-2HG or l-2HG, which is preferred over chemically synthesized racemic d,l-2HG. Beyond the occurrence as a product of cellular redox stress, l-2HG also plays a role in plants and eukaryotic cells as part of the mitochondrial metabolic repair mechanism (Hüdig et al., 2015). A side reaction of MDH yields small concentrations of l-2HG by reduction of 2-oxoglutarate. The mitochondrial FAD-containing oxidase l-2-hydroxyglutarate dehydrogenase (L2HGDH, EC 1.1.5.13) oxidizes l-2HG and the electrons produced in the reaction are transferred to the mitochondrial electron transport chain through the electron transfer protein (ETF) (Wanders et al., 1997; Rzem et al., 2004). An enzyme homologous to L2HGDH was also described in the model organism *Escherichia coli* (Knorr et al., 2018).

In bacteria, l-2HG mainly accumulates under carbon starvation conditions (Marschall et al., 1998; Metzner et al., 2004; Knorr et al., 2018). It is also an intermediate of the l-lysine degradation pathway in *Pseudomonadaceae* (Zhang et al., 2018; Thompson et al., 2019). Glutarate is the direct precursor of l-2HG. Hydroxylation of glutarate to l-2HG is catalyzed by a highly regio- and stereospecific Fe(II)/2-oxoglutarate-dependent dioxygenase CsiD (EC 1.14.11.64, also named glutarate hydroxylase) (Hibi and Ogawa, 2014). The co-product succinate is metabolized via the TCA-cycle. Since l-2-hydroxyglutarate oxidase (LghO) regenerates 2-oxoglutarate by oxidation of l-2HG (Knorr et al., 2018; Herr et al., 2019), the combined activities of CsiD and LghO convert the C5-dicarboxylic acid glutarate to the C4-dicarboxylic acid succinate. Thus, the absence of LghO is required for overproduction of l-2HG from glutarate.

Glutarate can be derived from l-lysine by four pathways that converge to 5AVA, which is converted to glutarate by GABA/5AVA aminotransferase (GabT) and the succinate/glutarate semialdehyde dehydrogenase (GabD). The first pathway from l-lysine to 5AVA employs l-lysine-α-oxidase (RaiP) from *Scomber japonicus* that catalyzes oxidative desamination of l-lysine using molecular oxygen followed by spontaneous decarboxylation (Cheng et al., 2020). The second pathway to 5AVA combines oxidative decarboxylation by l-lysine monooxygenase using molecular oxygen followed by desamidation by γ-aminovaleramidase from *P. putida* (Adkins et al., 2013). The third pathway is based on l-lysine decarboxylase from *E. coli*, putrescine oxidase PuO from *Rhodococcus qingshengii* and the γ-aminobutyraldehyde dehydrogenase from *E. coli* that catalyze decarboxylation, oxidative deamination using molecular oxygen and NAD-dependent oxidation (Haupka et al., 2020). The fourth pathway does not require molecular oxygen as it cascades l-lysine decarboxylase, 2-oxoglurate-dependent putrescine/cadaverine transaminase PatA, and NAD-dependent γ- aminobutyraldehyde dehydrogenase PatD from *E. coli* (Jorge et al., 2017). The pathway combinations for LdcC-PuO-PatD-GabT-GabD and LdcC-PatA-PatD-GabT-GabD couple conversion of lysine to glutarate in one or two transaminase reactions, respectively, that generate glutamate from 2-oxoglutarate. This coupling enabled flux enforcement, i.e., growth requires production of glutarate, which was achieved by deletion of *gdh*, the gene for the major ammonium assimilating enzyme l-glutamic acid dehydrogenase (Pérez-García et al., 2018; Haupka et al., 2020).

**Figure 1:**
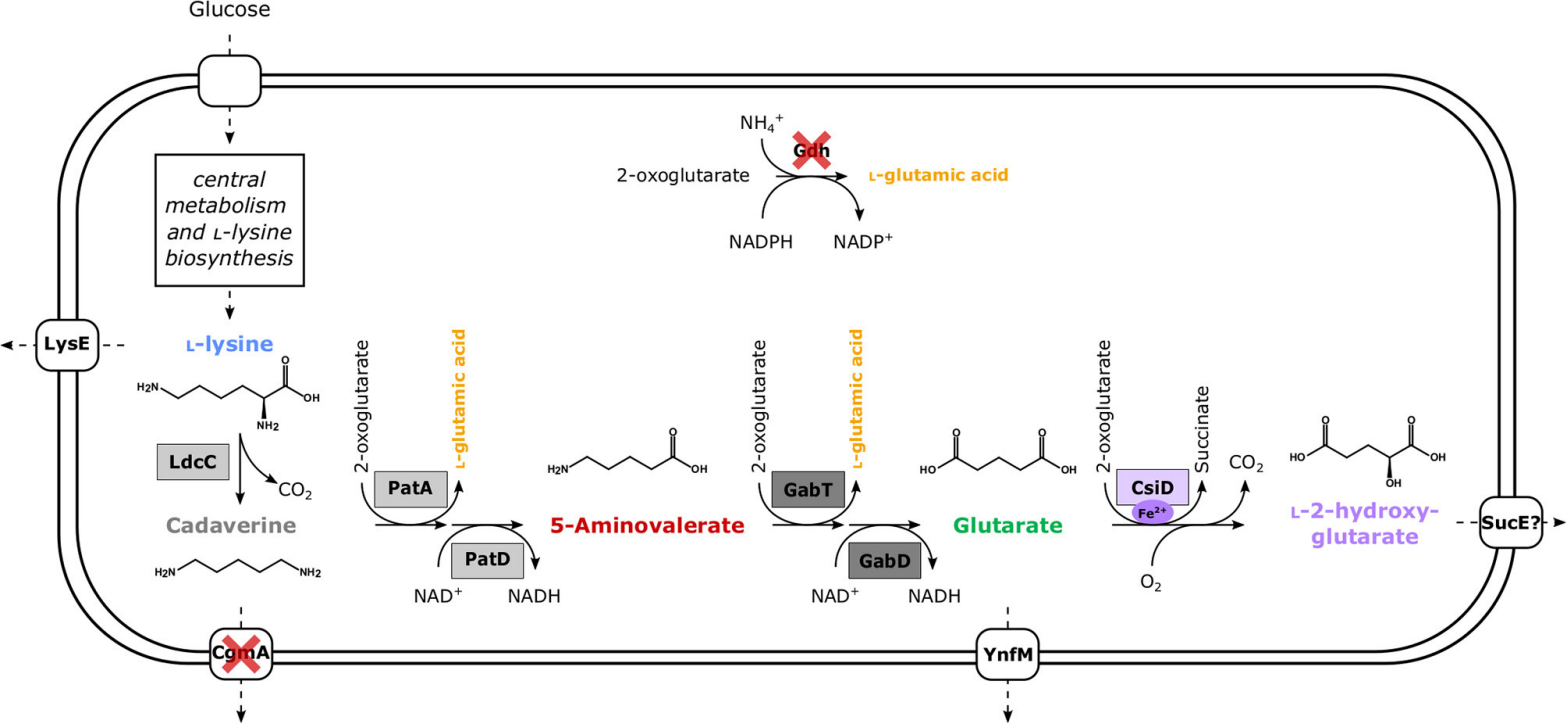
Schematic overview of metabolically engineered *C. glutamicum* overproducing l-2HG. Enzyme names are shown next to the reaction represented by the arrows. Dashed arrows represent several reaction steps. Heterologous enzymes are boxed, while gene deletions for enzymes are indicated by red crosses. Dark gray boxes depict enzymes encoded in *P. stutzeri* (*gabT*, GABA/5AVA amino transferase; *gabD*, succinate/glutarate-semialdehyde dehydrogenase) and light gray boxes those from *E. coli* (*ldcC*, l-lysine decarboxylase; *patA*, putrescine transaminase; *patD*, γ-aminobutyraldehyde dehydrogenase). In the case of glutarate hydroxylase (EC 1.14.1.64) CsiD enzymes (violet box) were sourced from either *E. coli* (HEGluA), *P. putida* (HPGluA), or *Halobacillus sp*. (HBGluA).

Since biosynthesis of l-2HG from glutarate requires molecular oxygen, glutarate biosynthesis from l-lysine by a pathway independent of molecular oxygen was extended by glutarate hydroxylase. The pathways RaiP-GabT-GabD, DavA-DavB-GabT-GabD, and LdcC-PuO-PatD-GabT-GabD involve oxygenases (RaiP, DavA, and PuO, respectively), whereas the pathway LdcC-PatA-PatD-GabT-GabD does not contain an enzyme using molecular oxygen as substrate (Jorge et al., 2017). To construct the six-step cascade LdcC-PatA-PatD-GabT-GabD-CsiD (Figure 1), CsiD enzymes from *E. coli* MG1655, *P. putida* KT2440 and *Halobacillus sp*. BA-2008 were tested since inspection of the genome sequence of *C. glutamicum* did not indicate that a CsiD homolog is encoded. The absence of LhgO from the *C. glutamicum* genome was considered beneficial since it indicated that l-2HG may not be degraded. This and the fact that we previously established efficient glutarate production employing the LdcC-PatA-PatD-GabT-GabD pathway provided an ideal starting point to establish fermentative production of l-2HG from renewable resources. This concept may be transferred to fermentative production of the stereoisomer d-2HG by extending the glutarate pathway with enzymes for conversion of glutarate to d-2HG.

## Materials and Methods

### Microorganisms and Cultivation Conditions

*E. coli* DH5α strain was used as a host for cloning (Hanahan, 1985), S17-1 for transconjugation (Schäfer et al., 1994). Both strains were grown in lysogeny broth (LB) at 37 °C and supplemented with antibiotics (50 μg mL^−1^ kanamycin, 100 μg mL^−1^ spectinomycin, 10 μg mL^−1^ tetracycline) when appropriate. *C. glutamicum* ATCC 13032 derived strains were cultivated in brain heart infusion with 0.5 M sorbitol (BHIS), or CGXII minimal medium (Eggeling and Bott, 2004) supplemented with 1 mM IPTG when appropriate. All bacterial strains and plasmids are listed in Tables 1, 2. For standard growth experiments in CGXII medium with *C. glutamicum*, overnight cultures in 10 mL BHIS were harvested and washed twice in TN-buffer (50 mM Tris-HCl, 50 mM NaCl, pH 6.3) before inoculation to an OD_600_ of 1 and supplementation with 40 g L^−1^ glucose as a sole carbon source. The cultivations in the BioLector microfermentation system (m2p-labs, Baesweiler, Germany) were performed in 3.2 mL FlowerPlates at 1,100 rpm with a filling volume of 1,000 μL.

**Table 1:** Bacterial strains used in this study.

**Strain**	**Relevant characteristics**	**References**
***E. coli***		
DH5α	Δ*lac*U169 (ϕ80*lac*Z ΔM15), *sup*E44, *hsd*R17, *rec*A1, *end*A1, *gyr*A96, *thi*-1, *rel*A1	Hanahan, 1985
S17-1	*recA, pro, hsdR*, RP4- 2Tc::Mu Km::Tn7 integrated into the chromosome	Simon et al., 1983
***C. glutamicum***		
WT	*C. glutamicum* ATCC13032	ATCC
GRLys1 (DM1933ΔCGP123)	*C. glutamicum* ATCC13032 with modifications: Δ*pck, pyc*^P458S^, *hom*^V59A^, 2 copies of *lysC*^T311I^, 2 copies of *asd*, 2 copies of *dapA*, 2 copies of *dapB*, 2 copies of *ddh*, 2 copies of *lysA*, 2 copies of *lysE*, in-frame deletion of prophages CGP1 (cg1507-cg1524), CGP2 (cg1746-cg1752) and CGP3 (cg1890-cg2071)	Unthan et al., 2015
GSLA2G	In-frame deletions of *sugR, ldhA, snaA* and *cgmA, gdh* in GRLys1	Pérez-García et al., 2018
GluA	GSLA2G(pVWEx1-*ldcC*) (pEKEx3-*patDA*) (pEC-XT99A-gabTD^*^)	Pérez-García et al., 2018
GluAΔ*sucE*	GSLA2GΔ*sucE* (pVWEx1-*ldcC*) (pEKEx3-*patDA*) (pEC-XT99A-gabTD^*^)	This study
GluA2	GSLA2GΔP*_*sucE*_*::P*_*tuf*_* (pVWEx1-*ldcC*)(pEKEx3-*patDA*)(pEC-XT99A-gabTD^*^)	This study
HEGluA	GSLA2G(pVWEx1-*ldcC-csiD^*Ec*^*) (pEKEx3-*patDA*) (pEC-XT99A-gabTD^*^)	This study
HBGluA	GSLA2G (pVWEx1-*ldcC-csiD^*Hb*^*) (pEKEx3-*patDA*) (pEC-XT99A-gabTD^*^)	This study
HPGluA	GSLA2G (pVWEx1-*ldcC-csiD^*Pp*^*)(pEKEx3-*patDA*)(pEC-XT99A-gabTD^*^)	This study
HPGluAΔ*sucE*	GSLA2GΔ*sucE* (pVWEx1-*ldcC-csiD^*Pp*^*) (pEKEx3-*patDA*) (pEC-XT99A-gabTD^*^)	This study
HPGluA2	GSLA2GΔP*_*sucE*_*::P*_*tuf*_* (pVWEx1-*ldcC-csiD^*Pp*^*) (pEKEx3-*patDA*) (pEC-XT99A-gabTD^*^)	This study

**Table 2:** Plasmids used in this study.

**Plasmid**	**Relevant characteristics**	**References**
pK19*mobsacB*	Kan^R^, mobilizable *E. coli* vector mutagenesis (*oriV, sacB*)	Schäfer et al., 1994
pK19*mobsacB*-Δ*sucE*	pK19*mobsacB* with a deletion construct *sucE*	This study
pK19*mobsacB*-ΔP*_*sucE*_*::P*_*tuf*_*	pK19*mobsacB* with a promoter exchange of *sucE* with the strong *tuf-*promoter and an optimized RBS	This study
pVWEx1	Kan^R^, *C. glutamicum*/*E. coli* shuttle vector (P_tac_, *lacI^*q*^*)	Peters-Wendisch et al., 2001
pVWEx1-*ldcC*	pVWEx1 expressing *ldcC* from *E.coli* MG1655	Pérez-García et al., 2018
pVWEx1-*ldcC-csiD^*Ec*^*	pVWEx1 expressing *ldcC* and *csiD* from *E.coli* MG1655	This study
pVWEx1-*ldcC-csiD^*Hb*^*	pVWEx1 expressing *ldcC* from *E.coli* MG1655 and *csiD* from *Halobacillus sp*. BA-2008	This study
pVWEx1-*ldcC-csiD^*Pp*^*	pVWEx1 expressing *ldcC* from *E.coli* MG1655 and *csiD* from *Pseudomonas putida* KT2440	This study
pEC-XT99A	Tet^R^, *C. glutamicum*/*E. coli* shuttle vector (P_trc_*, lacI^*q*^*, pGA1, o*riV_*Cg*_*)	Kirchner and Tauch, 2003
pEC-XT99A-gabTD^*^	pEC-XT99A expressing *gabT* and *gabD*^P134L^ from *P. stutzerii* ATCC17588	This study
pEKEx3	Spec^R^, *C. glutamicum*/*E. coli* shuttle vector (P_tac_ *lacI^*q*^* pBL1, o*riV_*Ec*_*)	Stansen et al., 2005
pEKEx3-*patDA*	pEKEx3, expressing *patD* and *patA* from *E. coli* MG1655	Pérez-García et al., 2018

*Km^R^: kanamycin resistance*.

*Spec^R^: spectomycin resistance*.

*Tet^R^: tetracycline resistance*.

To test for the utilization of l-2HG as carbon source, 12 mM l-2HG and 10 mM glucose were added, respectively, and the wildtype was inoculated to an initial OD_600_ of 0.5. For optimization of the iron concentration in CGXII minimal medium the respective concentrations of iron-(II)-sulfate (0–3 mM) were supplemented. To test the inhibitory effect of glutarate on l-2HG production, 20 or 40 mM of glutarate (pH 7.0) was added to CGXII medium, respectively.

For l-2HG production from wheat sidestream concentrate (WSC; obtained from Jäckering, Hamm, Germany) overnight cultures in 10 mL BHIS were harvested and washed twice in TN-buffer (50 mM Tris-HCl, 50 mM NaCl, pH 6.3) before inoculation to an OD_600_ of 1. The medium consisted of 246 g L^−1^ WSC, 20 g L^−1^ ammonium sulfate as nitrogen source, 42 g L^−1^ MOPS as buffer and 2 mM iron-(II)-sulfate (added as CsiD is an iron-containing enzyme). Growth in 10 mL Duetz microcultivation plates (Kuhner Shaker GmbH, Herzogenrath, Germany) was performed with different sandwich covers and culture volumes to alter the oxygen supply. Under “low oxygen” cultivation, cells were grown in 3 mL at 220 rpm in an Ecotron ET25-TA-RC (Infors HT, Einsbach, Germany) and plate sandwich covers for low evaporation (1.2 mm hole diameter) were used. For “high oxygen” supply the culture volume was decreased to 2 mL and standard plate sandwich covers (2.5 mm hole diameter) were used. Growth was monitored by determination of the OD_600_ with a V-1200 Spectrophotometer (VWR, Radnor, PA, USA).

### Molecular Biology Methods

Genomic DNA of *C. glutamicum, E. coli* and *P. putida* were isolated as described previously (Eikmanns et al., 1994). The gene *csiD* from *Halobacillus sp*. BA-2008 was codon-harmonized (Haupka, 2020) and synthesized with an optimized ribosomal binding site by Synbio Technologies (South Brunswick Township, New Jersey, United States of America). The standard molecular methods including plasmid isolation, molecular cloning and transformation of *E. coli* by heat shock and of *C. glutamicum* by electroporation with plasmid DNA were performed as described before (Eggeling and Bott, 2004). DNA sequences were amplified with the ALLin HiFi DNA Polymerase (HighQu, Kraichtal, Germany) using plasmid or genomic DNA as template. The oligonucleotides used in this study are listed in Table 3. The gene *ldcC* was amplified from the vector pVWEx1-*ldcC* (Pérez-García et al., 2018), whereas the different *csiD* genes were amplified from genomic DNA of the respective organisms and assembled together into BamHI-digested pVWEx1 by Gibson Assembly, using the respective primers. The constructed plasmids were transferred into *C. glutamicum* by transformation. For deletion, plasmid pK19*mobsacB* (Schäfer et al., 1994), digested with BamHI was assembled with amplified DNA fragments flanking the gene *sucE* (cg2425) using Gibson Assembly and was transferred into *E. coli* S17-1 to follow a protocol for gene deletion routinely applied (Eggeling and Bott, 2004). For replacement of the native promoter by the stronger *tuf-*promoter, the *tuf*-promoter and the flanking regions of the promoter of *sucE* were amplified from genomic DNA of *C. glutamicum* and assembled with the digested plasmid pK19*mobsacB*. For higher expression rates the start codon of *sucE* was changed from GTG to ATG and an optimized ribosome binding site (RBS) was included (Salis, 2011).

**Table 3:** Oligonucleotides used as primers in this study.

**Primer**	**Sequence (5′-3′)**	**Description**
ldcC-fw	CCTGCAGGTCGACTCTAGAGGATTCCGAAAGGAGGCCCTTCAGATGAACATCATTGCCATTATGGG	Construction of pVWEx1-*ldcC-csiD*
ldcC-csiD^Ec^-rv	CCTTTTGTATTCTTGTATTGGCGTTTTTATCCCGCCATTTTTAGG	
csiD^Ec^-fw	CCTAAAAATGGCGGGATAA*AAACGCCAATACAAGAATACAAAAGGAGGTAATTTT*ATGAATGCACTGACCGCCG	Construction of pVWEx1-*ldcC-csiD^*Ec*^*
csiD^Ec^-rv	*GAATTCGAGCTCGGTACCCGGGGAT*CTTACTGATGCGTCTGGTAGT	
ldcC-csiD^Pp^-rv	GTCCTGTTAACAGGACTAATTATAATTATCCCGCCATTTTTAGG	
csiD^Pp^-fw	CCTAAAAATGGCGGGATAA*TTATAATTAGTCCTGTTAACAGGACATCAAAGGAGGTTTTTTT*ATGAACGCCTTTACGCAG	Construction of pVWEx1-*ldcC-csiD^*Pp*^*
csiD^Pp^-rv	GAATTCGAGCTCGGTACCCGGGGATCTTATTGACCGCGCTGGTAC	
ldcC-csiD^Hb^-rv	*TTCCTTTAAGTTATACTTTCGTTAA*TTATCCCGCCATTTTTAGG	
csiD^Hb^-fw	CCTAAAAATGGCGGGATAA*TTAACGAAAGTATAACTTAAAGGAACCACGTATTT*ATGTGCGCAGTAGAAATG	Construction of pVWEx1-*ldcC-csiD^*Hb*^*
csiD^Hb^-rv	*GAATTCGAGCTCGGTACCCGGGGATC*CTATTGAAGGAATCGTCC	
ldcC-seq1	TGAACGATGTAGTGCCAGTC	
ldcC-seq2	GCAATGGGATTATTGCGTGG	Sequencing of pVWEx1-*ldcC-csiD*
ldcC-seq3	CAGGCAGAATCGAAGTGA	
csiD^Ec^-seq	CCGATTACGTGCTGATG	Sequencing of pVWEx1-*ldcC-csiD^*Ec*^*
csiD^Pp^-seq	GATCTGGTTCACGAAC	Sequencing of pVWEx1-*ldcC-csiD^*Pp*^*
csiD^Hb^-seq	GTAGGTTCCATCAGTG	Sequencing of pVWEx1-*ldcC-csiD^*Hb*^*
PSucEA	*GCATGCCTGCAGGTCGACTCTAGA*GGCGTGACGTGTACAAGCGCG	
PSucEB	TACGCGCCTACTGACACGCTAAAACTTAAGCCTCGCCCTTGCGTTC	Construction of pK19*mobsacB_*ΔP*_*sucE*_*::P*_*tuf*_*
P*_*tuf*_*-fw	GGCTGAACGCAAGGGCGAGGCTTAAGTTTTAGCGTGTCAGTAGGC	
P*_*tuf*_*-rv	AAGGAAGCTCAT*AAAAATACCTCCCCCAGTGTTCGTGCCGTCGCCCCGGCGACGAGTTTA*GTTACTGAATCCTAAGGGCA	
PSucEC	CGGCACGAACACTGGGGGAGGTATTTTT**A**TGAGCTTCCTTGTAGAAAATC	
PSucED	*AATTCGAGCTCGGTACCCGGGGATC*GAATAACGATGAGCACACCG	
PSucEE	GACTCGCTCACAAATGTGG	Verification of Promoter exchange ΔP*_*sucE*_*::P*_*tuf*_*
PSucEF	GAATTGCTCACCGTCTCG	
SucEA	*GCATGCCTGCAGGTCGACTCTAGAG*GTGGCACCTGGTGTTCCAG	
SucEB	TTTGGGCGGCCAGGATCTTTGCGATTTCTACAAGGAAGCTCAC	Construction of pK19*mobsacB_*Δ*sucE*
SucEC	GAATGGGTGAGCTTCCTTGTAGAAATCGCAAAGATCCTGGC	
SucED	*AATTCGAGCTCGGTACCCGGGGATC*CGAATGGATTGGTCAGGG	
SucEE	CTGCTGGTTGGGCTGTGG	Verification *sucE* deletion
SucEF	GTTAATCATGAGGCGTCG	Verification *sucE* deletion

*Overlaps to the vector are underlined, the ribosome binding site is indicated in italic and amino acid exchanges are shown in bold*.

### Coupled *in vitro* Activity of GabT and GabD

The apparent activities of GABA transaminase GabT and succinate semialdehyde oxidoreductase GabD were assayed in combination by monitoring NADPH formation after the addition of 2-oxoglutarate. The preparation of the crude extract was carried out as previously described (Pérez-García et al., 2018). The 1 mL assay mix contained 150 mM phosphate buffer (pH 9.0), l-2HG (2, 4, 8, 12 mM) or NaCl as control, 0.1 mM pyridoxal-5′-phosphate, 1 mM NADP^+^, 20 mM 5AVA, and 0.5 mg mL^−1^ crude extract. The reaction was started by the addition of 15 mM 2-oxoglutatarate. Protein concentrations were determined with the Bradford assay kit (Bio-Rad Laboratories, Hercules, CA, United States) using BSA (bovine serum albumin) as standard. The formation of NADPH was monitored photometrically at 340 nm and 30°C for 3 min using a Shimadzu UV-1202 spectrophotometer (Shimadzu, Duisburg, Germany).

### Quantification of Amino Acids, Diamines, and Carboxylic Acids

The quantification of extracellular amino acids and their derivatives, carbohydrates and carboxylic acids in the cultivation medium was performed with a high-performance liquid chromatography system (1200 series, Agilent Technologies Deutschland GmbH, Böblingen, Germany). After centrifugation of 1 mL of cell cultures at 14,000 rpm for 10 min the supernatant was stored at −20°C prior to analysis. Analysis of l-lysine, 5AVA and the diamine cadaverine was performed by an automatic pre-column derivatization with *ortho*-phthaldialdehyde (OPA) and separated on a reversed phase HPLC using pre- and main column (LiChrospher 100 RP8 EC-5μ, 125 × 4.6 mm, CS Chromatographie Service GmbH) with l-asparagine as internal standard (Schneider and Wendisch, 2010). Detection of the fluorescent derivatives was carried out with a fluorescence detector with an excitation wavelength of 230 nm and an emission wavelength of 450 nm. Glutarate and l-2HG and glucose concentrations were measured with an amino exchange column (Aminex, 300 × 8 mm, 10 μm particle size, 25 Å pore diameter, CS Chromatographie Service GmbH) under isocratic conditions as described previously with a flow of 0.8 mL min^−1^ (Schneider et al., 2011). The substances were detected with a refractive index detector (RID G1362A, 1200 series, Agilent Technologies) and a diode array detector (DAD G1315B, 1200 series, Agilent Technologies) at 210 nm.

### Fermentative Production

A baffled bioreactor with total a volume of 3.6 L was used (KLF, Bioengineering AG, Switzerland). Three six-bladed Rushton turbines were placed on the stirrer axis with a distance from the bottom of the reactor of 6, 12, and 18 cm. The aspect ratio of the reactor was 2.6:1.0 and the stirrer to reactor diameter ratio was 0.39. Automatic control of the stirrer speed between 400 and 1500 rpm kept the relative dissolved oxygen saturation at 30%. A constant airflow of 1 or 2 NL min^−1^ was maintained from the bottom through a sparger, corresponding to an aeration of 0.5 and 1 vvm, respectively.

The pH was kept constant at 7.0 ± 0.1 by automatic addition of phosphoric acid [10% (*v/v*)] and potassium hydroxide (4 M). The temperature was maintained at 30°C. To prevent foaming 0.6 mL L^−1^ of the antifoam agent AF204 (Sigma Aldrich, Darmstadt, Germany) was added and a mechanical foam breaker was present to serve as an additional foam control. The fermentation was performed with a head space overpressure of 0.2 bar. The initial working volume of 2 L was inoculated to an OD_600_ of 1.5 from a shake flask pre-culture in CGXII minimal medium supplemented with 40 g L^−1^ glucose, 1 mM IPTG and 2 mM FeSO_4_. Samples were collected by an autosampler and cooled down to 4°C until further use. The feed consisted only of 600 g L^−1^ glucose (ρ = 1.21 kg m^−3^) and was started 4 h after the cells reached the late stationary phase, indicated by the pO_2_ rising above 80% again after the initial growth phase. The glucose feed was applied for 5 min with a flow of 1.2 mL min^−1^ when the pO_2_ surpassed 60%. Further feed was only added, when the pO2 decreased to 30% after the addition of the feed solution to prevent oversaturation with glucose.

## Results

### Establishing *de novo* Biosynthesis of l-2HG by *C. glutamicum*

**Figure 2:**
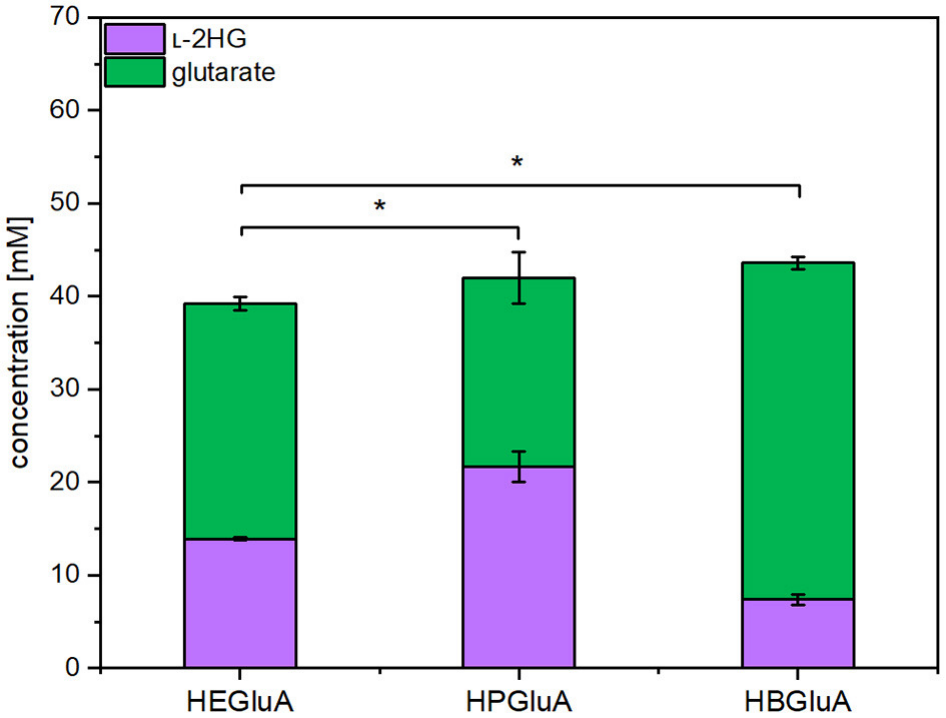
Product titers of l-2HG and its precursor glutarate obtained with strains overproducing different glutarate hydroxylases. *C. glutamicum* strains HEGluA, HPGluA, and HBGluA expressing *csiD* from *E. coli* MG1655, *P. putida* KT2440, and *Halobacillus sp*. BA-2008, respectively, were grown in 40 g L^−1^ glucose CGXII minimal medium supplemented with 1 mM IPTG in the microcultivation device BioLector. Values and error bars represent means and standard deviations from 3 replicate cultivations with supernatants analyzed after 96 h. Statistical significance was assessed by Student's paired *t*-testing (*p < 0.05, n.s, not significant).

As *C. glutamicum* can utilize some organic acids (Wendisch et al., 2016), the response of *C. glutamicum* WT to l-2HG was determined. When l-2HG was added to mineral salts medium as sole carbon source instead of glucose, no growth of *C. glutamicum* was observed. Moreover, when l-2HG plus glucose were assayed, *C. glutamicum* WT utilized glucose, but did not degrade l-2HG as revealed by HPLC analysis of the culture supernatants (data not shown). Thus, l-2HG does neither serve as sole nor as combined carbon source for *C. glutamicum*, which can be considered as a suitable host for production of l-2HG.

l-2HG is not a known metabolite of *C. glutamicum*, but it has been engineered for overproduction of glutarate (Pérez-García et al., 2018). Inspection of the genome of *C. glutamicum* did not indicate the presence of a gene coding for an enzyme hydroxylating glutarate. In some bacteria like *E. coli* (Marschall et al., 1998; Knorr et al., 2018; Herr et al., 2019), *P. putida*, and *Halobacillus sp*. (Thompson et al., 2019), glutarate hydroxylase, also known as glutarate dioxygenase (EC 1.14.11.64), uses molecular oxygen to hydroxylate glutarate to l-2HG with concomitant decarboxylation of 2-oxoglutarate to succinate. Thus, the respective *csiD* genes from *E. coli* MG1655, *P. putida* KT2440 and *Halobacillus sp*. BA-2008 were overexpressed in synthetic operons with *ldcC* from *E. coli*. The resulting pVWEx1-plasmids were used to transform *C. glutamicum* GSLA2G (pEKEx3_*patDA*) (pEC-XT99A_gabTD*). The resulting strains were named HEGluA, HPGluA, and HBGluA, respectively. In production experiments, the three strains produced and secreted l-2HG. After 96 h *C. glutamicum* strain HEGluA accumulated 14 ± 0 mM l-2HG, strain HBGluA 7 ± 1 mM l-2HG, and strain HPGluA 22 ± 2 mM l- 2HG (Figure 2). The strains showed comparable maximal growth rates of 0.13 ± 0.00 h^−1^, but strain HPGluA showed a lag-phase of 24 h. Taken together, a proof-of-principle for production of l-2HG by *C. glutamicum* was demonstrated.

### Role of the Succinate Exporter SucE in Export of Glutarate and l-2HG

**Figure 3:**
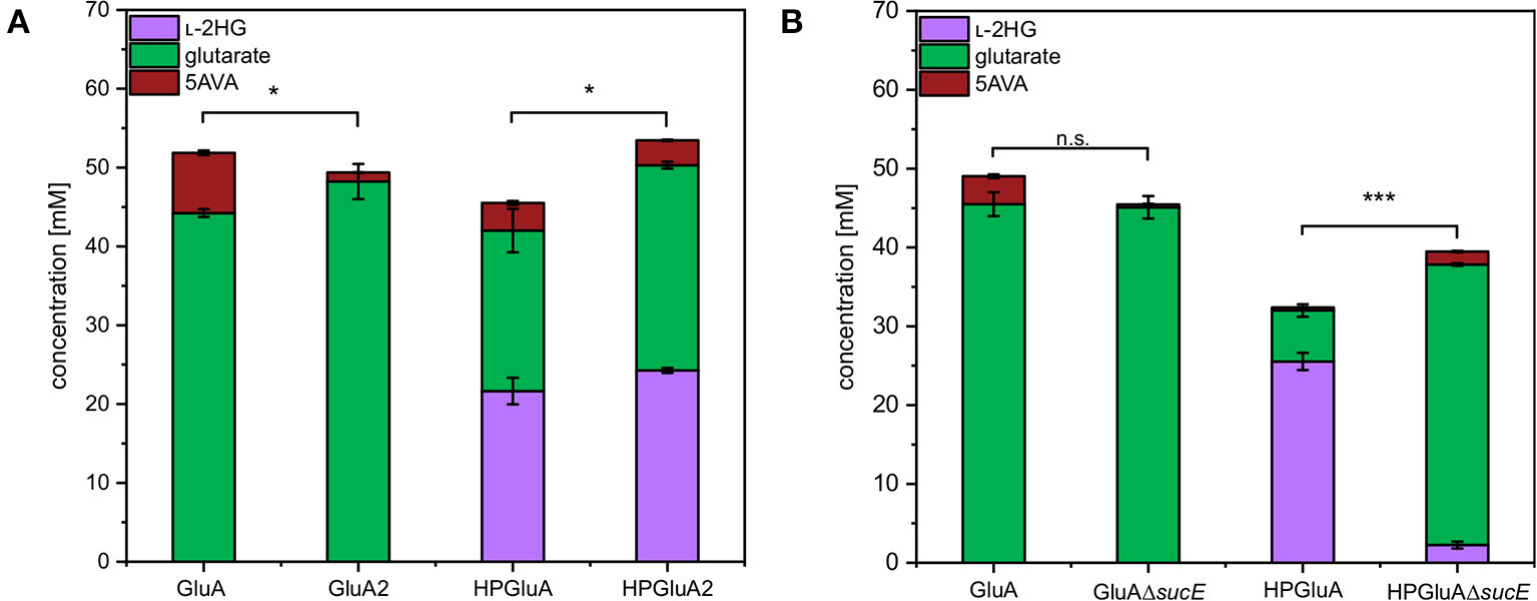
Influence of overexpression and deletion of *sucE* on production of l-2HG, glutarate and 5AVA. **(A)** Strains GluA2 and HPGluA2 differed from strains GluA and HPGluA by overexpression of *sucE*. **(B)** Strains GluAΔ*sucE* and HPGluAΔ*sucE* were derived from strains GluA and HPGluA, respectively, by deletion of *sucE*. Strains were grown in the BioLector using 40 g L^−1^ glucose minimal medium supplemented with 1 mM IPTG and supernatants were analyzed after 120 h. Values and error bars represent mean and standard deviation values (*n* = 3 cultivations). Statistical significance was assessed in Student's paired *t*-test (***p < 0.001, *p < 0.05, n.s. not significant).

SucE is known to play a role in the export of the dicarboxylic acid succinate out of the *C. glutamicum* cell (Huhn et al., 2011). Since l-2HG and glutarate are also dicarboxylic acids, the role of SucE in the export of l-2HG and glutarate was studied. To this end, the *sucE* gene was either overexpressed by replacing the native promoter with the stronger *tuf*-promoter or deleted. The glutarate producing strain GluA2 that overexpressed *sucE* produced significantly more glutarate than the control strain GluA (44 ± 0 vs. 48 ± 2 mM; Figure 3A). Similarly, upon overexpression of *sucE*, production of l-2HG was slightly elevated (24 ± 0 mM for HPGluA2 vs. 22 ± 2 mM for HPGluA; Figure 3A) as well as production of the by-product glutarate (26 ± 0 mM for HPGluA2 vs. 20 ± 3 mM for HPGluA; Figure 3A). Thus, overexpression of *sucE* was beneficial for production of glutarate as well as l-2HG.

Deletion of *sucE* in glutarate producer GluA did not negatively affect glutarate production, however, growth was slowed (0.12 ± 0.00 h^−1^ for GluA vs. 0.07 ± 0.00 h^−1^ for GluAΔ*sucE*; Figure 3B). Thus, SucE is not the main export system for glutarate and at least one other export system is able to compensate for the lack of SucE regarding glutarate export. Importantly, deletion of *sucE* reduced production of l-2HG more than 10-fold (2 ± 0 mM for HPGluAΔ*sucE* vs. 26 ± 1 mM for HPGluA; Figure 3B), while the growth rate was not significantly impacted (0.09 ± 0.00 h^−1^ for HPGluA vs. 0.08 ± 0.01 h^−1^ for HPGluAΔ*sucE*). The findings that *sucE* overexpression positively affected l-2HG production and that deletion of *sucE* dramatically reduced l-2HG production suggested that SucE may be active as export system for l-2HG.

### Enhanced Conversion of Glutarate to l-2HG by Increased Iron Concentrations

**Figure 4:**
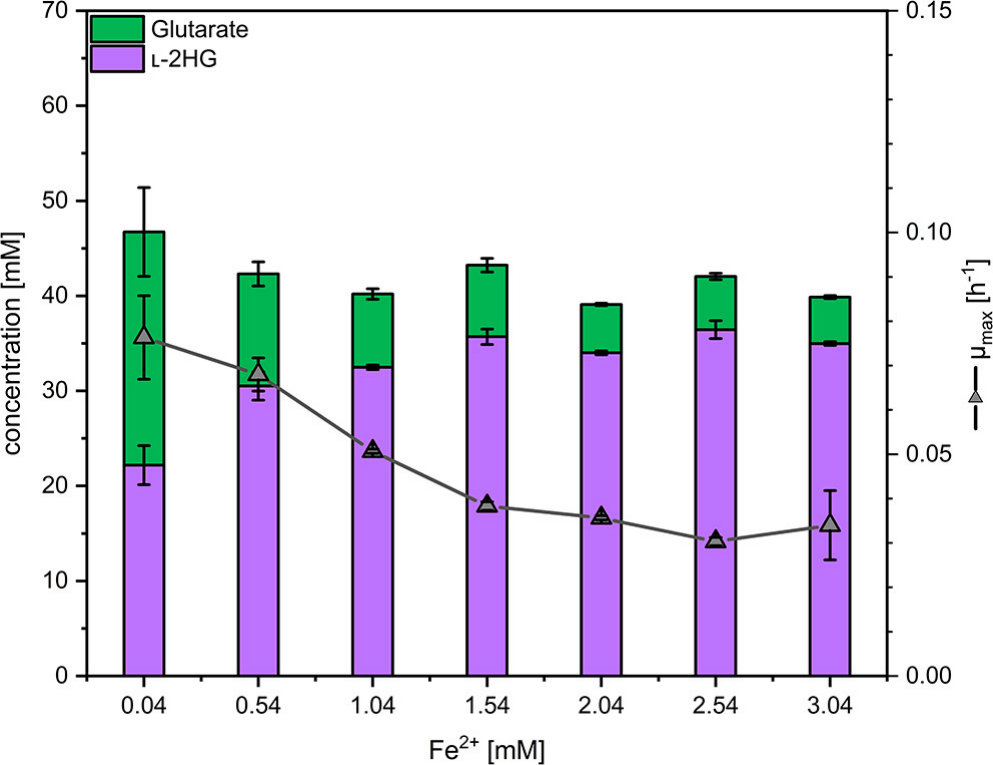
Influence of the iron concentration on the maximal growth rate and production of l-2HG and glutarate. *C. glutamicum* HPGluA2 was grown in the BioLector with 40 g L^−1^ glucose minimal medium supplemented with 1 mM IPTG and the indicated iron concentrations. Supernatants were analyzed after 96 h. Values and error bars represent means and standard deviations (*n* = 3 cultivations).

As the glutarate hydroxylase CsiD is an Fe^2+^-dependent metalloenzyme (Herr et al., 2019; Thompson et al., 2019), the concentration of iron (II) in the media was varied and the effect on production of l-2HG determined. Concentrations of 0.5 to 3 mM FeSO_4_ were added on top of the standard concentration (0.037 mM) in CGXII minimal medium (Eggeling and Bott, 2004). Three trends were observed: with increasing iron concentrations the growth rate and the formation of glutarate as by-product were reduced, while production of l-2HG was significantly increased (Figure 4). At an iron concentration of 2.04 mM, 5 ± 0 mM glutarate and 34 ± 0 mM l-2HG accumulated in the supernatant (Figure 4), thus, about 87% of glutarate were converted to l-2HG, while at the standard iron concentration l-2HG and glutarate accumulated to about equimolar concentrations. The growth inhibitory effect of elevated iron concentrations may be due to increased production of l-2HG and/or other effects elicited by higher iron concentrations. Thus, 2.04 mM was choosen as the optimal iron concentration for further experiments to find the best compromise between l-2HG titer and volumetric productivity.

### Inhibitory Effects on Key Enzymes in l-2HG Pathway

**Figure 5:**
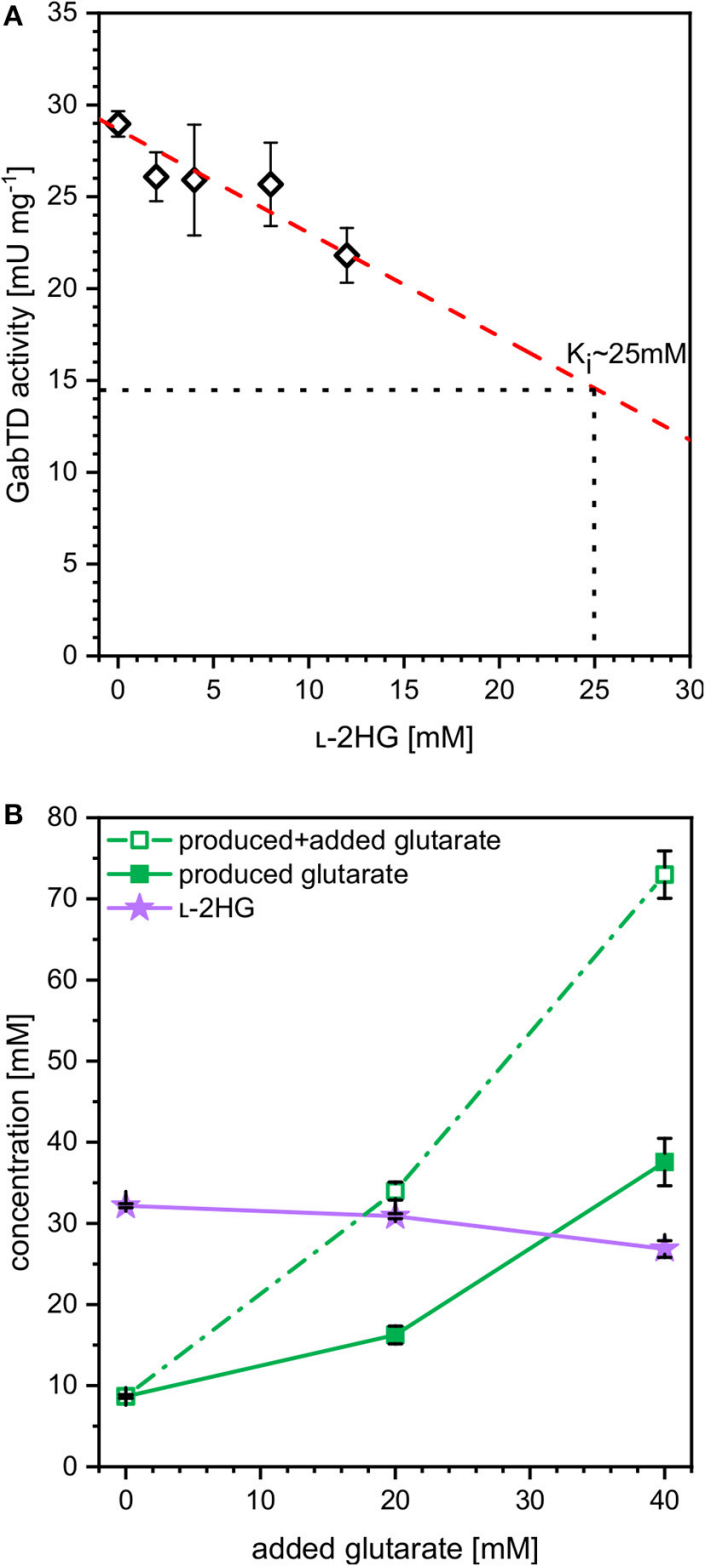
Influence of l-2HG on the combined *in vitro* enzyme activities of GABA transaminase GabT and succinate semialdehyde dehydrogenase GabD **(A)** and influence of extracellularly added glutarate on production of l-2HG **(B)**. **(A)** Crude extracts of GluA were assayed for combined *in vitro* enzyme activities of GABA transaminase GabT and succinate semialdehyde dehydrogenase GabD in the presence of increasing concentrations of l-2HG. **(B)** Strain HPGluA2 was cultivated in the BioLector with 40 g L^−1^ glucose minimal medium supplemented with 1 mM IPTG, 2 mM iron (II)-sulfate and increasing concentrations of glutarate (0, 20, 40 mM). Supernatant concentrations of l-2HG (filled violet triangles), glutarate (open green squares) as well as the net glutarate concentrations produced in addition to the added glutarate concentration (closed green squares) were determined after 96 h and are given as means and standard deviations of three independent cultivations.

The finding that at higher iron concentrations less glutarate and more l-2HG accumulated, while the combined concentration of glutarate plus l-2HG was highest at the lowest iron concentration indicated bottlenecks. These may arise due to inhibition of glutarate hydroxylase CsiD by its product l-2HG (Knorr et al., 2018) and/or due to inhibition of enzymes of glutarate biosynthesis. The transaminases PatA from *E. coli* and GabT from *P. stutzerii* are crucial for glutarate biosynthesis (Pérez-García et al., 2018) and some transaminases are known to be inhibited by l-2HG, which blocks binding of the substrate 2-oxoglutarate (McBrayer et al., 2018). To approach this problem, enzyme activity assays were performed with crude extracts of *C. glutamicum* GluA. A combined activity assay of GABA transaminase GabT and succinate semialdehyde dehydrogenase GabD was performed as described previously (Pérez-García et al., 2018). In the presence of increasing concentrations of l-2HG (0–12 mM) the combined GabT and GabD activity was reduced. By extrapolation, it was determined that 25 mM l-2HG resulted in half-maximal GabTD activity (Figure 5A). Thus, l-2HG negatively affects production of its precursor glutarate.

In order to determine if increased glutarate concentrations are beneficial for production of l-2HG, production of l-2HG was determined in the absence or presence of extracellularly added glutarate (0, 20, or 40 mM). Notably, externally added glutarate slightly reduced production of l-2HG, whereas glutarate accumulation was increased (Figure 5B). When 0, 20, or 40 mM glutarate were added to the medium before inoculation, additional glutarate was produced: 9, 16, and 38 mM, respectively (Figure 5B). Thus, extracellular glutarate addition exerts a positive effect on glutarate production. The slight reduction of l-2HG production upon addition of extracellular glutarate may be due to substrate inhibition of glutarate hydroxylase CsiD.

### l-2HG Production at Bioreactor Scale

**Figure 6:**
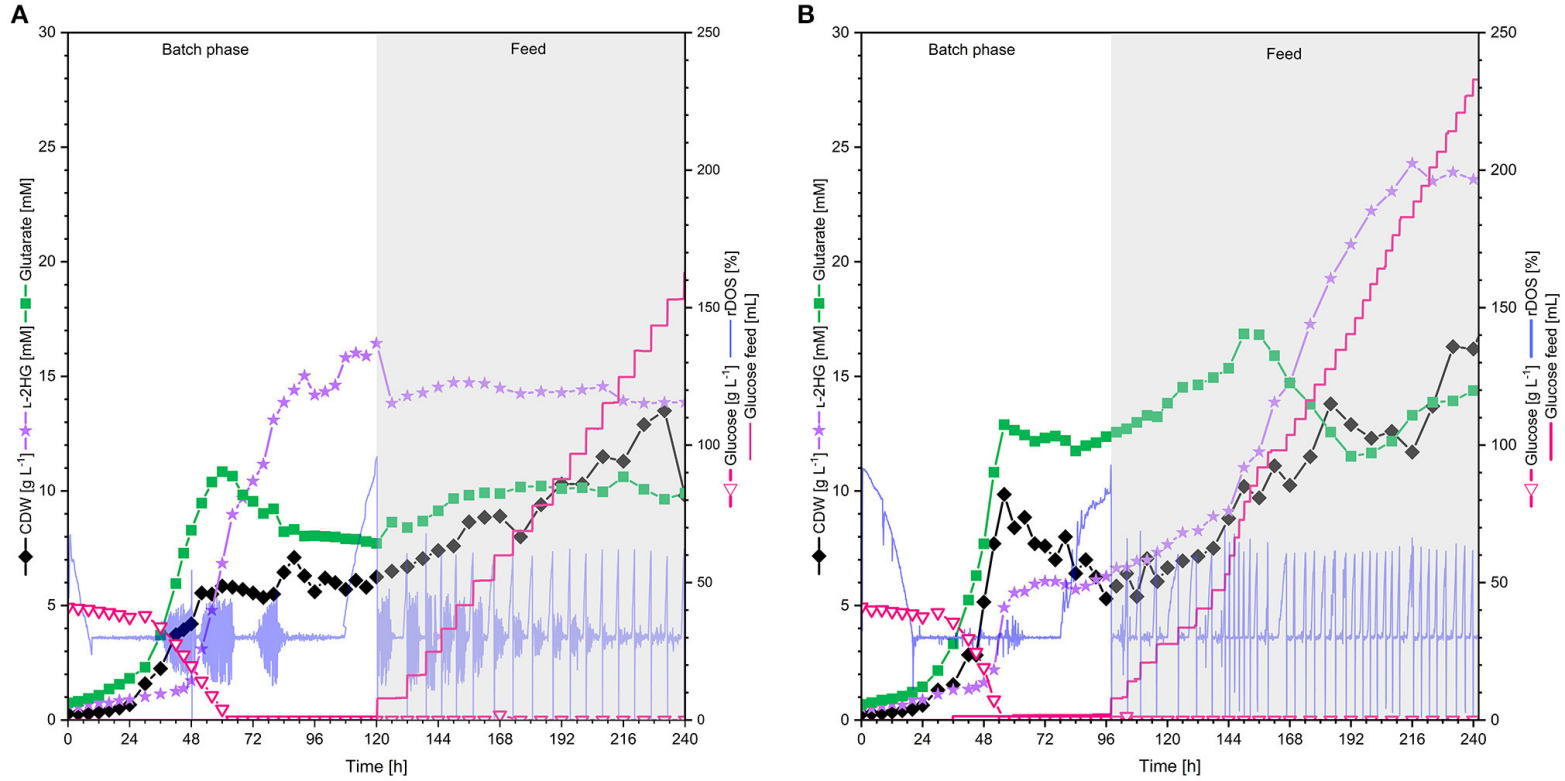
l-2HG production by *C. glutamicum* HPGluA2 in fed-batch fermentation with **(A)** 0.5 vvm and **(B)** 1 vvm aeration rate. HPGluA2 was cultivated in CGXII minimal medium in fed-batch mode over 240 h, containing 40 g L^−1^ glucose and feeding 600 g L^−1^ glucose solution. l-2HG concentration is indicated in violet stars (mM), biomass concentration (CDW) is shown in black diamands (g L^−1^), glucose concentration (g L^−1^) is plotted as pink hollow triangles, and glutarate concentration (mM) in green squares, 600 g L^−1^ glucose feed (mL) is plotted as pink line and the relative dissolved oxygen saturation (rDOS) is indicated in light blue (%). Cultivation was performed at 30°C and pH 7.0 regulated with 10% (*v/v*) H_3_PO_4_ and 4 M KOH. An overpressure of 0.2 bar was applied. 0.6 mL L^−1^ of antifoam agent AF204 (Sigma Aldrich, Taufkirchen, Germany) was added to the medium manually before inoculation.

In order to test if l-2HG production by strain HPGluA2 is stable at larger volumes, 2 L scale bioreactor fermentations were performed. Two aeration rates were tested: 0.5 and 1 vvm (Figure 6). Under both conditions cells grew with similar growth rates (0.5 vvm: 0.07 h^−1^, 1 vvm: 0.08 h^−1^) and displayed a similar entry into the stationary phase after 56 h. Oscillations in rDOS was due to overregulation of the stirrer, an often observed disadvantage of PID controllers, especially, when combined with the rDOS probes that are highly sensitive to fluctuations. However, almost 2-fold more biomass was formed at the higher aeration rate (0.5 vvm: 6.3 g L^−1^, 1 vvm: 9.9 g L^−1^). Accumulation of the precursor glutarate was growth-coupled and ceased upon entry into the stationary phase (titers of around 10 mM at 0.5 vvm and 13 mM at 1 vvm), whereas production of l-2HG was delayed and started after 30 h. While l-2HG production at an aeration rate of 0.5 vvm reached the highest titer of 16 mM at 120 h (Figure 6A), l-2HG production at an aeration rate of 1 vvm stopped at a titer of 6 mM shortly after glucose was fully depleted (Figure 6B). Thus, the latter fermentation was switched to feeding mode to supply the culture with more carbon source. As consequence, the strain grew to a 2-fold higher biomass concentration of 16.3 g L^−1^ and produced fourfold more l-2HG reaching a titer of 24 mM (Figure 6B).

Comparing the different cultivations in batch mode (Table 4) it becomes obvious that the l-2HG product yield showed an inverse relationship with the aeration rate, as its production was highest in the not actively aerated microcultivation system. Although HPGluA2 grew 2-fold slower in the microcultivation system, the volumetric productivity was higher than in the bioreactors (Table 4). By contrast, growth proceeded to the highest biomass concentration and the highest biomass yield was observed when aeration was highest (Table 4).

**Table 4:** Comparison of l-2HG process parameters during different cultivation strategies.

**System**	**Mode/Phase**	**Aeration** **rate** **[vvm]**	**μ_max_** **[h^−1^]**	**CDW** **[g L^−1^]**	**Titer** **[g L^−1^]**	**Y**_X/S_ **[g g^−1^]**	**Y**_P/S_ **[g g^−1^]**	**VP** **[g L^−1^ h^−1^]**
Micro-Cultivation	Batch	–	0.04	4.3	5.0	0.11	0.13	0.05
Bioreactor	Batch	0.5	0.07	6.3	2.4	0.16	0.06	0.02
	Batch	1	0.08	9.9	0.9	0.25	0.02	0.01
	Fed-batch	1	0.07	16.3	3.5	0.09	0.02	0.01

*μ_max_, maximal growth rate; CDW, cell dry weight; Y_X/S_, biomass yield; Y_P/S_, product yield; VP, volumetric productivity*.

### l-2HG Production From Wheat Sidestream Concentrate

**Figure 7:**
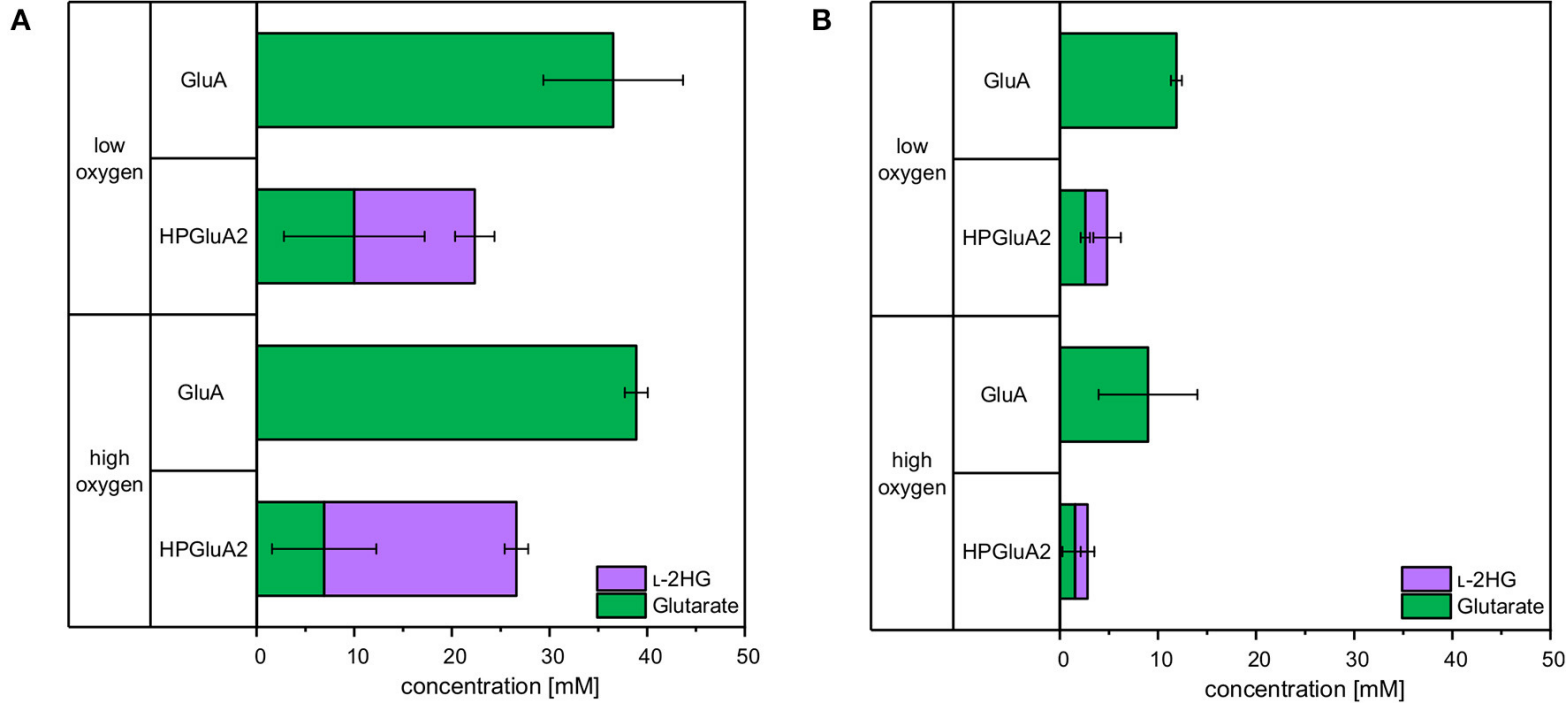
Comparison of glutarate and l-2HG production based on glucose **(A)** or wheat sidestream concentrate **(B)**. *C. glutamicum* glutarate producer GluA and l-2HG producer HPGluA2 were grown in the Duetz microcultivation plates with low or high oxygen supply using covers of different air permeability and different volumes (3 and 2 mL). Strains were cultivated in CGXII minimal medium with 40 g L^−1^ glucose or with a mixture containing 246 g L^−1^ WSC, 20 g L^−1^ ammonium sulfate and 42 g L^−1^ MOPS. Both media were supplemented with 1 mM IPTG and 2 mM FeSO_4_. Supernatants were analyzed after 96 h. Values and error bars represent means and standard deviations of 3 cultivations.

Sustainable processes based on renewable feedstock are sought after. Therefore, it was tested if l-2HG can be produced from a sidestream of industrial starch production (wheat sidestream concentrate; WSC). WSC contains hardly any starch, but various sugars like glucose, fructose, sucrose, raffinose, xylose and arabinose (D'Appolonia and Rayas-Duarte, 1994). Production of glutarate from WSC was also analyzed. To this end, *C. glutamicum* strains GluA and HPGluA2 were cultivated for 96 h either in CGXII medium with glucose or in WSC medium. Aeration was altered by different cultivation volumes and by using either “low oxygen” or “high oxygen” Duetz plates. For HPGluA2 both media were supplemented with 2 mM FeSO_4_. Glucose-based glutarate production by strain GluA was comparable for both aeration schemes (37 ± 7 and 39 ± 1 mM; Figure 7), whereas it was lower in WSC medium (12 ± 1 and 9 ± 5 mM of glutarate under high and low oxygen supply, respectively; Figure 7). HPLC analysis of WSC using a refractive index detector did not allow to identify all peaks, but we could show that around 12 mM glucose and 6 mM maltose were utilized (data not shown) and 3 ±0 mM glutarate and 2 ± 1 mM l-2HG accumulated under low oxygen conditions. No significant differences with respect to l-2HG production by strain HPGluA2 with different oxygen supply were observed (Figure 7). Albeit leading to lower titers, production of l-2HG to concentrations around 2 mM were achieved. Thus, l-2HG production from the renewable feedstock wheat sidestream concentrate was demonstrated.

## Discussion

In this study, production of the sought after compound l-2HG by the industrial workhorse *C. glutamicum* was demonstrated after extension of lysine biosynthesis in a six-step cascade employing the metalloenzyme CsiD from *P. putida* as final step. The glucose-based process was stable in 2 L bioreactor cultivations and a l-2HG titer of 3.5 g L^−1^ was obtained in fed-batch fermentation. Moreover, l-2HG production based on the renewable feedstock wheat sidestream concentrate was demonstrated.

l-2HG production was achieved by glutarate hydroxylase extending lysine biosynthesis in a six-step cascade, in which only the last committed step catalyzed by oxygen-dependent glutarate hydroxylase requires oxygen. Glutarate hydroxylases belong to the large family of non-heme Fe(II)- and 2-oxoglutarate-dependendent oxygenases, which are essential for diverse biological functions. These enzymes form an Fe(IV)-oxo intermediate to initiate oxidative transformations and can be assigned to four major types of reactions: hydroxylation, halogenation, ring formation and desaturation (Hausinger, 2004). The glutarate hydroxylases which have been studied in this research belong to the enzymes catalyzing a hydroxylation reaction at an unactivated carbon center by incorporation of molecular oxygen (Guengerich, 2015; Martinez and Hausinger, 2015). The CsiD enzymes from *E. coli* MG1655 and *P. putida* KT2440 have been recently characterized in different studies displaying their high specificities toward the native substrate glutarate with K_M_-values around 0.65 mM in *E. coli* (Knorr et al., 2018) and around 0.15 mM for *P. putida* (Zhang et al., 2018). The K_M_-values of around 0.1 mM for the co-substrate 2-oxoglutarate are comparable for both enzymes. The higher affinity of glutarate hydroxylase from *P. putida* for glutarate is reflected by the better conversion of glutarate to l-2HG observed in this study. Even though no K_M_ value for the glutarate hydroxylase from *Halobacillus sp*. BA-2008 has been determined yet, it was demonstrated that it converts 5 mM glutarate to l-2HG with comparable efficiency to the other tested hydroxylases with 2-oxoglutarate as co-substrate (Thompson et al., 2019). Although the capability of CsiD from *Halobacillus sp*. BA-2008 to produce l-2HG from glutarate was demonstrated in *C. glutamicum*, the efficiency of the codon harmonized version of CsiD was inferior compared to the enzymes derived from other organisms.

It could be demonstrated that l-2HG inhibits the combined GabTD activity with an inhibitory constant of about 25 mM (Figure 5A). Due to its structural similarity to 2-OG, l-2HG potentially inhibits transamination reactions (McBrayer et al., 2018) by competitive inhibition (Intlekofer et al., 2015). Here, possibly competitive inhibition of 2-OG dependent transaminase GabT by l-2HG may have limited l-2HG product titers. Potentially, this may be overcome by enzyme engineering of the 2-OG binding pocket of GabT for better differentiation of this substrate from l-2HG. We have chosen enzymes GabT and GabD from *P. stutzeri* as they performed better than those from *C. glutamicum, P. putida* and *P. syringae* regarding glutarate production (Pérez-García et al., 2018). Since we did not compare GabT and GabD enzymes from various sources for inhibition of their activities by l-2HG, identifying feedback resistant enzymes/variants of the GabT and GabD enzymes in future work may help to increase l-2HG production. On the other hand, glutarate hydroxylase CsiD is subject to weak product inhibition (Knorr et al., 2018). Therefore, CsiD from other sources or variants may be selected that exhibit no or reduced product inhibition in order to improve l-2HG production.

Moreover, a weak substrate inhibition on l-2HG production by glutarate could also be identified (Figure 5B). CsiD from *E. coli* was recently characterized and the reaction mechanism was described (Knorr et al., 2018; Herr et al., 2019). Since the substrate analogon *N*-oxalylglycine (NOG), a 2-OG mimic, inhibited CsiD (Knorr et al., 2018), this feedback inhibition may limit l-2HG production. However, since we did not assay feedback inhibition of various glutarate hydroxylases by l-2HG, future work to improve l-2HG production should involve the identification of feedback resistant CsiD enzymes/variants.

Surprisingly, extracellular addition of glutarate boosted glutarate production. This is unlikely due to effects on enzyme activities. *C. glutamicum* possesses chromosomal copies of *gabT* and *gabD* (Pérez-García et al., 2018; Haupka et al., 2020). The PucR-like regulator GabR that requires GABA as coactivator activates transcription of the *gabTDP* operon (Zhu et al., 2020). Possibly, glutarate mimics GABA as coactivator of GabR activating the endogenous *gabTDP* operon and increasing GabT and GabD enzyme levels in addition to plasmid-borne expression of *gabT* and *gabD* from *P. stutzeri*.

Cultivation schemes with higher oxygen supply reduced l-2HG production, but improved growth to high biomass concentrations. Glutarate hydroxylases are 2-OG-dependent enzymes and these metalloenzymes specifically require Fe^2+^ as cofactor for their function (Mitchell et al., 2017; Dunham et al., 2018). Previously, it has been shown that increased iron concentrations improve enzyme activity (Fukumori and Hausinger, 1993). Increased Fe^2+^ concentrations improved l-2HG product titers, but slowed growth significantly. This might be due to iron effects such as toxicity mainly due to the formation of hydroxyl radicals as oxidative stress response (Braun, 1997; Touati, 2000; Eid et al., 2017) and/or due to inhibition of 2-OG-dependent dioxygenases and methylases, which play an essential role in DNA/RNA repair (van den Born et al., 2009), by l-2HG (Low et al., 2001; Ledesma-García et al., 2016). Provision of oxygen and iron to the *C. glutamicum* cell are interlinked. Molecular oxygen is required for 2-OG-dependent dioxygenases such as CsiD to form the Fe(IV)-oxo intermediate, but with too much oxygen decoupling occurs via “non-productive reactions” resulting in inactive Fe(III)-containing enzymes (Hausinger, 2004) as observed with AlkB (Henshaw et al., 2004) and TfdA (Liu et al., 2001). Secondly, an “uncoupled turnover” of the co-substrate 2-OG may occur by decomposition of 2-OG to carbon dioxide and succinate. These “uncoupled reactions” might contribute to the lower titers of l-2HG obtained in 2 L scale fermentative production with the highest oxygen supply. The addition of ascorbic acid is a promising option since ascorbate improved hydroxylase activity (Fukumori and Hausinger, 1993). Alternatively, production of l-2HG may be improved by concomitant overproduction of either glutathione (Liu et al., 2019) or ascorbate in *C. glutamicum*.

Our results suggested that export of l-2HG involves SucE (Figure 3). Surprisingly, glutarate export does not depend solely on SucE. Overexpression of *sucE* increased succinate production (Zhu et al., 2014) similar to increased production of glutarate and l-2HG observed here. Deletion of *sucE* revealed that SucE is the main export system for l-2HG, but not for glutarate. Production of l-2HG may benefit from re-uptake of glutarate secreted to the culture medium as by-product. However, the uptake system for glutarate is unknown. MctC is involved in the uptake of pyruvate, propionate, and acetate, but it is not clear if it accepts glutarate (Jolkver et al., 2009).

Glutarate was observed as significant by-product. Unlike l-2HG, which likely is exported by SucE (s. above), glutarate is exported by YnfM (Fukui et al., 2019). Overexpression of *ynfM* improved production of glutarate, succinate, and 2-OG (Fukui et al., 2019; Han et al., 2020). Thus, deletion of *ynfM* is a suitable strategy to abolish export of glutarate as by-product of l-2HG production.

Alternative feedstocks are important to achieve sustainable biotechnological processes. In this respect, sidestreams are highly relevant. Glycerol is a sidestream of the biodiesel process and glycerol accumulates as stoichiometric by-product. *C. glutamicum* has been engineered to produce amino acids from pure and technical grade glycerol (Rittmann et al., 2008; Meiswinkel et al., 2013b). Access to nitrogenous sidestreams from the fishery industry such as glucosamine or *N*-acetyl-glucosamine is important if the target product is containing nitrogen atoms (Matano et al., 2014, 2016). For products lacking nitrogen atoms, access to lignocellulosics is pivotal (Gopinath et al., 2011; Buschke et al., 2013; Meiswinkel et al., 2013a; Hadiati et al., 2014; Matsuura et al., 2019). The focus of this study was a sidestream of industrial starch production. Although production was lower compared to glucose (30% for glutarate, 15% for l-2HG), media composition was easy and required only a buffer, a nitrogen source for growth and iron for glutarate hydroxylase. We envision improved production of l-2HG from wheat sidestream concentrate once access to sugars other than the native substrates glucose, fructose, maltose, and sucrose is achieved, e.g., to raffinose, xylose and arabinose, that are present in this feedstock (D'Appolonia and Rayas-Duarte, 1994). Strategies for access to the lignocellulosic pentoses xylose and arabinose have been established (Kawaguchi et al., 2008; Gopinath et al., 2011; Schneider et al., 2011; Meiswinkel et al., 2013a; Imao et al., 2017) and may prove useful for improved production of l-2HG from the alternative feedstock wheat sidestream concentrate. In this respect, provision of iron and/or iron chelators may prove essential as shown here for CsiD function.

The approach described here may be followed for production of the stereoisomer d-2HG. To this end, the glutarate pathway described here has to be extended with enzymes for conversion of glutarate to d-2HG. These enzymes may be sourced from lysine degrading bacteria as d-2HG occurs as intermediate in some of these pathways, e.g., in *E. coli* (Zhao and Winkler, 1996) and different *Pseudomonaceace* (Zhang et al., 2017; Guo et al., 2018; Thompson et al., 2019). Future work will reveal if this approach is suitable for fermentative production of d-2HG.

## Data Availability Statement

The original contributions presented in the study are included in the article/supplementary material, further inquiries can be directed to the corresponding author/s.

## Author's Note

VFW wishes to dedicate this article in memoriam of scientific and personal friend Michael M. Goodin, Professor of Plant Pathology, University of Kentucky, Lexington, KY.

## Author Contributions

CP and VFW conceived and designed the experiments. CP constructed plasmids and strains, cultivated, and analyzed *C. glutamicum* strains and prepared a draft of the manuscript. CP and FM performed bioreactor experiments and evaluated data. AB conducted the growth experiment on wheat sidestream concentrate. CP, AB, FM, and VFW finalized the manuscript. VFW acquired funding and coordinated the study. All authors read and approved the final version of the manuscript.

## Conflict of Interest

The authors declare that the research was conducted in the absence of any commercial or financial relationships that could be construed as a potential conflict of interest.

## References

[B1] Adkins J. Jordan J. Nielsen D. R. (2013). Engineering *Escherichia coli* for renewable production of the 5-carbon polyamide building-blocks 5-aminovalerate and glutarate. Biotechnol. Bioeng. 110, 1726–1734. 10.1002/bit.24828 23296991

[B2] Braun V. (1997). Avoidance of iron toxicity through regulation of bacterial iron transport. Biol. Chem. 378, 779–786. 9377472

[B3] Buschke N. Becker J. Schäfer R. Kiefer P. Biedendieck R. Wittmann C. (2013). Systems metabolic engineering of xylose-utilizing *Corynebacterium glutamicum* for production of 1,5-diaminopentane. Biotechnol. J. 8, 557–570. 10.1002/biot.201200367 23447448

[B4] Cheng J. Luo Q. Duan H. Peng H. Zhang Y. Hu J.. Efficient whole-cell catalysis for 5-aminovalerate production from L-lysine by using engineered *Escherichia coli* with ethanol pretreatment. Sci Rep. (2020) 10, 990. 10.1038/s41598-020-57752-x 31969619 PMC6976619

[B5] Chowdhury R. Yeoh K. K. Tian Y. Hillringhaus L. Bagg E. A. Rose N. R.. (2011). The oncometabolite 2-hydroxyglutarate inhibits histone lysine demethylases. EMBO Rep. 12, 463–469. 10.1038/embor.2011.43 21460794 PMC3090014

[B6] D'Appolonia B. L. Rayas-Duarte P. (1994). Wheat carbohydrates: structure and functionality, in Wheat: Production, Properties and Quality, eds Bushuk W. Rasper V. F. (Boston, MA: Springer), 107–127. 10.1007/978-1-4615-2672-8_8

[B7] Dunham N. P. Chang W. Mitchell A. J. Martinie R. J. Zhang B. Bergman J. A.. (2018). Two distinct mechanisms for C–C desaturation by iron(II)- and 2-(Oxo)glutarate-dependent oxygenases: importance of α-heteroatom assistance. J. Am. Chem. Soc. 140, 7116–7126. 10.1021/jacs.8b01933 29708749 PMC5999578

[B8] Eggeling L. Bott M. (2004). Handbook of *Corynebacterium glutamicum, 1st Edn*. Bota Raton, FL: CRC Press; LLC.

[B9] Eid R. Arab N. T. T. Greenwood M. T. (2017). Iron mediated toxicity and programmed cell death: a review and a re-examination of existing paradigms. Biochim. Biophys. Acta Mol. Cell Res. 1864, 399–430. 10.1016/j.bbamcr.2016.12.002 27939167

[B10] Eikmanns B. J. Thum-Schmitz N. Eggeling L. Lüdtke K. U. Sahm H. (1994). Nucleotide sequence, expression and transcriptional analysis of the *Corynebacterium glutamicum gltA* gene encoding citrate synthase. Microbiology 140, 1817–1828. 10.1099/13500872-140-8-1817 7522844

[B11] Fukui K. Nanatani K. Nakayama M. Hara Y. Tokura M. Abe K. (2019). *Corynebacterium glutamicum* CgynfM encodes a dicarboxylate transporter applicable to succinate production. J. Biosci. Bioeng. 127, 465–471. 10.1016/j.jbiosc.2018.10.004 30392965

[B12] Fukumori F. Hausinger R. P. (1993). Purification and characterization of 2,4-dichlorophenoxyacetate/alpha-ketoglutarate dioxygenase. J. Biol. Chem. 268, 24311–24317. 8226980

[B13] Gopinath V. Meiswinkel T. M. Wendisch V. F. Nampoothiri K. M. (2011). Amino acid production from rice straw and wheat bran hydrolysates by recombinant pentose-utilizing *Corynebacterium glutamicum*. Appl. Microbiol. Biotechnol. 92, 985–996. 10.1007/s00253-011-3478-x 21796382

[B14] Guengerich F. P. (2015). Introduction: metals in biology: α-ketoglutarate/iron-dependent dioxygenases. J. Biol. Chem. 290, 20700–20701. 10.1074/jbc.R115.675652 26152720 PMC4543631

[B15] Guo X. Zhang M. Cao M. Zhang W. Kang Z. Xu P.. (2018). d-2-Hydroxyglutarate dehydrogenase plays a dual role in l-serine biosynthesis and d-malate utilization in the bacterium *Pseudomonas stutzeri*. J. Biol. Chem. 293, 15513–15523. 10.1074/jbc.RA118.003897 30131334 PMC6177604

[B16] Hadiati A. Krahn I. Lindner S. N. Wendisch V. F. (2014). Engineering of *Corynebacterium glutamicum* for growth and production of L-ornithine, L-lysine, and lycopene from hexuronic acids. Bioresour. Bioprocess. 1:25 10.1186/s40643-014-0025-5

[B17] Han T. Kim G. B. Lee S. Y. (2020). Glutaric acid production by systems metabolic engineering of an l-lysine–overproducing *Corynebacterium glutamicum*. Proc. Natl. Sci. U.S.A. 117, 30328–30334. 10.1073/pnas.2017483117 33199604 PMC7720191

[B18] Hanahan D. (1985). Techniques for transformation of *E. coli*. DNA Cloning A Practical Approach 1, 109–135.

[B19] Haupka C. (2020). Chaupka/Codon_Harmonization: Release v1.2.0. 10.5281/zenodo.4062177

[B20] Haupka C. Delépine B. Irla M. Heux S. Wendisch V. F. (2020). Flux enforcement for fermentative production of 5-aminovalerate and glutarate by *Corynebacterium glutamicum*. Catalysts 10:1065 10.3390/catal10091065

[B21] Hausinger R. P. (2004). Fe(II)/α-ketoglutarate-dependent hydroxylases and related enzymes. Crit. Rev. Biochem. Mol. Biol. 39, 21–68. 10.1080/10409230490440541 15121720

[B22] Henshaw T. F. Feig M. Hausinger R. P. (2004). Aberrant activity of the DNA repair enzyme AlkB. J. Inorg. Biochem. 98, 856–861. 10.1016/j.jinorgbio.2003.10.021 15134932

[B23] Herr C. Q. Macomber L. Kalliri E. Hausinger R. P. (2019). Glutarate L-2-hydroxylase (CsiD/GlaH) is an archetype Fe(II)/2-oxoglutarate-dependent dioxygenase. Adv. Protein Chem. Struct. Biol. 117, 63–90. 10.1016/bs.apcsb.2019.05.001 31564307 PMC7132994

[B24] Hibi M. Ogawa J. (2014). Characteristics and biotechnology applications of aliphatic amino acid hydroxylases belonging to the Fe(II)/α-ketoglutarate-dependent dioxygenase superfamily. Appl. Microbiol. Biotechnol. 98, 3869–3876. 10.1007/s00253-014-5620-z 24682483

[B25] Hüdig M. Maier A. Scherrers I. Seidel L. Jansen E. E. W. Mettler-Altmann T.. (2015). Plants possess a cyclic mitochondrial metabolic pathway similar to the mammalian metabolic repair mechanism involving malate dehydrogenase and l-2-hydroxyglutarate dehydrogenase. Plant Cell Physiol. 56, 1820–1830. 10.1093/pcp/pcv108 26203119

[B26] Huhn S. Jolkver E. Krämer R. Marin K. (2011). Identification of the membrane protein SucE and its role in succinate transport in *Corynebacterium glutamicum*. Appl. Microbiol. Biotechnol. 89, 327–335. 10.1007/s00253-010-2855-1 20809072

[B27] Imao K. Konishi R. Kishida M. Hirata Y. Segawa S. Adachi N.. (2017). 1,5-Diaminopentane production from xylooligosaccharides using metabolically engineered *Corynebacterium glutamicum* displaying beta-xylosidase on the cell surface. Bioresour. Technol. 245, 1684–1691. 10.1016/j.biortech.2017.05.135 28599919

[B28] Intlekofer A. M. Dematteo R. G. Venneti S. Finley L. W. S. Lu C. Judkins A. R.. (2015). Hypoxia induces production of L-2-hydroxyglutarate. Cell Metab. 22, 304–311. 10.1016/j.cmet.2015.06.023 26212717 PMC4527873

[B29] Intlekofer A. M. Wang B. Liu H. Shah H. Carmona-Fontaine C. Rustenburg A. S.. (2017). L-2-Hydroxyglutarate production arises from noncanonical enzyme function at acidic pH. Nat. Chem. Biol. 13, 494–500. 10.1038/nchembio.2307 28263965 PMC5516644

[B30] Jolkver E. Emer D. Ballan S. Krämer R. Eikmanns B. J. Marin K. (2009). Identification and characterization of a bacterial transport system for the uptake of pyruvate, propionate, and acetate in *Corynebacterium glutamicum*. J. Bacteriol. Res. 191, 940–948. 10.1128/JB.01155-08 19028892 PMC2632059

[B31] Jorge J. M. P. Pérez-García F. Wendisch V. F. (2017). A new metabolic route for the fermentative production of 5-aminovalerate from glucose and alternative carbon sources. Bioresour. Technol. 245, 1701–1709. 10.1016/j.biortech.2017.04.108 28522202

[B32] Kawaguchi H. Sasaki M. Vertès A. A. Inui M. Yukawa H. (2008). Engineering of an L-arabinose metabolic pathway in *Corynebacterium glutamicum*. Appl. Microbiol. Biotechnol. 77, 1053–1062. 10.1007/s00253-007-1244-x 17965859

[B33] Kirchner O. Tauch A. (2003). Tools for genetic engineering in the amino acid-producing bacterium *Corynebacterium glutamicum*. J. Biotechnol. 104, 287–299. 10.1016/S0168-1656(03)00148-2 12948646

[B34] Knorr S. Sinn M. Galetskiy D. Williams R. M. Wang C. Müller N.. (2018). Widespread bacterial lysine degradation proceeding via glutarate and L-2-hydroxyglutarate. Nat. Commun. 9:5071. 10.1038/s41467-018-07563-6 30498244 PMC6265302

[B35] Ledesma-García L. Sánchez-Azqueta A. Medina M. Reyes-Ramírez F. Santero E. (2016). Redox proteins of hydroxylating bacterial dioxygenases establish a regulatory cascade that prevents gratuitous induction of tetralin biodegradation genes. Sci. Rep. 6:23848. 10.1038/srep23848 27030382 PMC4814904

[B36] Liu A. Ho R. Y. N. Que L. Ryle M. J. Phinney B. S. Hausinger R. P. (2001). Alternative reactivity of an α-ketoglutarate-dependent iron(II) oxygenase: enzyme self-hydroxylation. J. Am. Chem. Soc. 123, 5126–5127. 10.1021/ja005879x 11457355

[B37] Liu W. Zhu X. Lian J. Huang L. Xu Z. (2019). Efficient production of glutathione with multi-pathway engineering in *Corynebacterium glutamicum*. J. Ind. Microbiol. Biotechnol. 46, 1685–1695. 10.1007/s10295-019-02220-3 31420796

[B38] Low D. A. Weyand N. J. Mahan M. J. (2001). Roles of DNA adenine methylation in regulating bacterial gene expression and virulence. Infect. Immun. 69, 7197–7204. 10.1128/IAI.69.12.7197-7204.2001 11705888 PMC98802

[B39] Marschall C. Labrousse V. Kreimer M. Weichart D. Kolb A. Hengge-Aronis R. (1998). Molecular analysis of the regulation of *csiD*, a carbon starvation-inducible gene in *Escherichia coli* that is exclusively dependent on σS and requires activation by cAMP-CRP. J. Mol. Biol. 276, 339–353. 10.1006/jmbi.1997.1533 9512707

[B40] Martinez S. Hausinger R. P. (2015). Catalytic mechanisms of Fe(II)- and 2-oxoglutarate-dependent oxygenases. J. Biol. Chem. 290, 20702–20711. 10.1074/jbc.R115.648691 26152721 PMC4543632

[B41] Matano C. Kolkenbrock S. Hamer S. N. Sgobba E. Moerschbacher B. M. Wendisch V. F. (2016). *Corynebacterium glutamicum* possesses β-N-acetylglucosaminidase. BMC Microbiol. 16:177. 10.1186/s12866-016-0795-3 27492186 PMC4974736

[B42] Matano C. Uhde A. Youn J.-W. Maeda T. Clermont L. Marin K.. (2014). Engineering of *Corynebacterium glutamicum* for growth and l-lysine and lycopene production from N-acetyl-glucosamine. Appl. Microbiol. Biotechnol. 98, 5633–5643. 10.1007/s00253-014-5676-9 24668244

[B43] Matsuura R. Kishida M. Konishi R. Hirata Y. Adachi N. Segawa S.. (2019). Metabolic engineering to improve 1,5-diaminopentane production from cellobiose using β-glucosidase-secreting *Corynebacterium glutamicum*. Biotechnol. Bioeng. 116, 2640–2651. 10.1002/bit.27082 31184369

[B44] McBrayer S. K. Mayers J. R. DiNatale G. J. Shi D. D. Khanal J. Chakraborty A. A.. (2018). Transaminase inhibition by 2-hydroxyglutarate impairs glutamate biosynthesis and redox homeostasis in glioma. Cell 175, 101–116.e25. 10.1016/j.cell.2018.08.038 30220459 PMC6219629

[B45] Meiswinkel T. M. Gopinath V. Lindner S. N. Nampoothiri K. M. Wendisch V. F. (2013a). Accelerated pentose utilization by *Corynebacterium glutamicum* for accelerated production of lysine, glutamate, ornithine and putrescine. Microb. Biotechnol. 6, 131–140. 10.1111/1751-7915.12001 23164409 PMC3917455

[B46] Meiswinkel T. M. Rittmann D. Lindner S. N. Wendisch V. F. (2013b). Crude glycerol-based production of amino acids and putrescine by *Corynebacterium glutamicum*. Bioresour. Technol. 145, 254–258. 10.1016/j.biortech.2013.02.053 23562176

[B47] Metzner M. Germer J. Hengge R. (2004). Multiple stress signal integration in the regulation of the complex σS-dependent *csiD-ygaF-gabDTP* operon in *Escherichia coli*. Mol. Microbiol. 51, 799–811. 10.1046/j.1365-2958.2003.03867.x 14731280

[B48] Mitchell A. J. Dunham N. P. Martinie R. J. Bergman J. A. Pollock C. J. Hu K.. (2017). Visualizing the reaction cycle in an iron(II)- and 2-(oxo)-glutarate-dependent hydroxylase. J. Am. Chem. Soc. 139, 13830–13836. 10.1021/jacs.7b07374 28823155 PMC5852378

[B49] Nan A. Feher I. C. (2017). A new polyester based on allyl α-hydroxy glutarate as shell for magnetite nanoparticles. AIP Conf. Proc. 1917:040003 10.1063/1.5018285

[B50] Navarro E. Subirana J. A. Puiggali J. (1997). The structure of nylon 12,5 is characterized by two hydrogen bond directions as are other polyamides derived from glutaric acid. Polymer 38, 3429–3432. 10.1016/S0032-3861(97)00017-7

[B51] Oldham W. M. Clish C. B. Yang Y. Loscalzo J. (2015). Hypoxia-mediated increases in l-2-hydroxyglutarate coordinate the metabolic response to reductive stress. Cell Metab. 22, 291–303. 10.1016/j.cmet.2015.06.021 26212716 PMC4526408

[B52] Pan B.-C. Chen W.-H. Lee T.-M. Liou G.-S. (2018). Synthesis and characterization of novel electrochromic devices derived from redox-active polyamide–TiO2 hybrids. J. Mater. Chem. C 6, 12422–12428. 10.1039/C8TC04469D

[B53] Pérez-García F. Jorge J. M. P. Dreyszas A. Risse J. M. Wendisch V. F. (2018). Efficient production of the dicarboxylic acid glutarate by *Corynebacterium glutamicum* via a novel synthetic pathway. Front. Microbiol. 9:2589. 10.3389/fmicb.2018.02589 30425699 PMC6218589

[B54] Peters-Wendisch P. Schiel B. Wendisch V. F. Katsoulidis E. Möckel B. Sahm H.. (2001). Pyruvate carboxylase is a major bottleneck for glutamate and lysine production by *Corynebacterium glutamicum*. J. Mol. Microbiol. Biotechnol. 3, 295–300. Available online at: https://www.caister.com/backlist/jmmb/v/v3/v3n2/22.pdf 11321586

[B55] Rittmann D. Lindner S. N. Wendisch V. F. (2008). Engineering of a glycerol utilization pathway for amino acid production by *Corynebacterium glutamicum*. Appl. Environ. Microbiol. 74, 6216–6222. 10.1128/AEM.00963-08 18757581 PMC2570274

[B56] Rzem R. Veiga-da-Cunha M. Noel G. Goffette S. Nassogne M.-C. Tabarki B.. (2004). A gene encoding a putative FAD-dependent l-2-hydroxyglutarate dehydrogenase is mutated in L-2-hydroxyglutaric aciduria. Proc. Natl. Acad. Sci. U.S.A. 101, 16849–16854. 10.1073/pnas.0404840101 15548604 PMC534725

[B57] Salis H. M. (2011). Chapter two - the ribosome binding site calculator, in Methods in Enzymology Synthetic Biology, Part B, ed Voigt C. (Cambridge, MA: Academic Press), 19–42. 10.1016/B978-0-12-385120-8.00002-4 21601672

[B58] Schäfer A. Tauch A. Jäger W. Kalinowski J. Thierbach G. Pühler A. (1994). Small mobilizable multi-purpose cloning vectors derived from the *Escherichia coli* plasmids pK18 and pK19: selection of defined deletions in the chromosome of *Corynebacterium glutamicum*. Gene 145, 69–73. 10.1016/0378-1119(94)90324-7 8045426

[B59] Schneider J. Niermann K. Wendisch V. F. (2011). Production of the amino acids l-glutamate, l-glutamate, l-lysine, l-ornithine and l-arginine from arabinose by recombinant *Corynebacterium glutamicum*. J. Biotechnol. 154, 191–198. 10.1016/j.jbiotec.2010.07.009 20638422

[B60] Schneider J. Wendisch V. F. (2010). Putrescine production by engineered *Corynebacterium glutamicum*. Appl. Microbiol. Biotechnol. 88, 859–868. 10.1007/s00253-010-2778-x 20661733

[B61] Shelar S. Shim E.-H. Brinkley G. J. Kundu A. Carobbio F. Poston T.. (2018). Biochemical and epigenetic insights into L-2-hydroxyglutarate, a potential therapeutic target in renal cancer. Clin. Cancer Res. 24, 6433–6446. 10.1158/1078-0432.CCR-18-1727 30108105 PMC6295227

[B62] Shim E.-H. Livi C. B. Rakheja D. Tan J. Benson D. Parekh V.. (2014). L-2-Hydroxyglutarate: an epigenetic modifier and putative oncometabolite in renal cancer. Cancer Discov. 4, 1290–1298. 10.1158/2159-8290.CD-13-0696 25182153 PMC4286872

[B63] Simon R. Priefer U. Pühler A. (1983). A broad host range mobilization system for *in vivo* genetic engineering: transposon mutagenesis in gram negative bacteria. Bio/Technology 1, 784–791. 10.1038/nbt1183-784

[B64] Stansen C. Uy D. Delaunay S. Eggeling L. Goergen J. L. Wendisch V. F. (2005). Characterization of a *Corynebacterium glutamicum* lactate utilization operon induced during temperature-triggered glutamate production. Appl. Environ. Microbiol. 71, 5920–5928. 10.1128/AEM.71.10.5920-5928.2005 16204505 PMC1265975

[B65] Thompson M. G. Blake-Hedges J. M. Cruz-Morales P. Barajas J. F. Curran S. C. Eiben C. B.. (2019). Massively parallel fitness profiling reveals multiple novel enzymes in *Pseudomonas putida* lysine metabolism. mBio 10:e02577–18. 10.1128/mBio.02577-18 31064836 PMC6509195

[B66] Touati D. (2000). Iron and oxidative stress in bacteria. Arch. Biochem. Biophys. 373, 1–6. 10.1006/abbi.1999.1518 10620317

[B67] Unthan S. Baumgart M. Radek A. Herbst M. Siebert D. Brühl N.. (2015). Chassis organism from Corynebacterium glutamicum-a top-down approach to identify and delete irrelevant gene clusters. Biotechnol. J. 10, 290–301. 10.1002/biot.201400041 25139579 PMC4361050

[B68] van den Born E. Bekkelund A. Moen M. N. Omelchenko M. V. Klungland A. Falnes P. Ø. (2009). Bioinformatics and functional analysis define four distinct groups of AlkB DNA-dioxygenases in bacteria. Nucleic. Acids Res. 37, 7124–7136. 10.1093/nar/gkp774 19786499 PMC2790896

[B69] Varela O. Orgueira H. A. (2000). Synthesis of chiral polyamides from carbohydrate-derived monomers, in Advances in Carbohydrate Chemistry and Biochemistry, ed Horton D. (Cambridge, MA: Academic Press), 137–174. 10.1016/S0065-2318(00)55005-7

[B70] Wanders R. J. A. Vilarinho L. Hartung H. P. Hoffmann G. F. Mooijer P. A. W. Jansen G. A.. (1997). L-2-hydroxyglutaric aciduria: normal L-2-hydroxyglutarate dehydrogenase activity in liver from two new patients. J. Inherit. Metab. Dis. 20, 725–726. 10.1023/A:1005355316599 9323578

[B71] Wendisch V. F. Brito L. F. Gil Lopez M. Hennig G. Pfeifenschneider J. Sgobba E.. (2016). The flexible feedstock concept in industrial biotechnology: metabolic engineering of *Escherichia coli, Corynebacterium glutamicum, Pseudomonas, Bacillus* and yeast strains for access to alternative carbon sources. J. Biotechnol. 234, 139–157. 10.1016/j.jbiotec.2016.07.022 27491712

[B72] Xu W. Yang H. Liu Y. Yang Y. Wang P. Kim S.-H.. (2011). Oncometabolite 2-hydroxyglutarate is a competitive inhibitor of α-ketoglutarate-dependent dioxygenases. Cancer Cell 19, 17–30. 10.1016/j.ccr.2010.12.014 21251613 PMC3229304

[B73] Zhang M. Gao C. Guo X. Guo S. Kang Z. Xiao D.. (2018). Increased glutarate production by blocking the glutaryl-CoA dehydrogenation pathway and a catabolic pathway involving l-2-hydroxyglutarate. Nat. Commun. 9:2114. 10.1038/s41467-018-04513-0 29844506 PMC5974017

[B74] Zhang W. Zhang M. Gao C. Zhang Y. Ge Y. Guo S.. (2017). Coupling between d-3-phosphoglycerate dehydrogenase and d-2-hydroxyglutarate dehydrogenase drives bacterial l-serine synthesis. Proc. Natl. Acad. Sci. U.S.A. 114, E7574–E7582. 10.1073/pnas.1619034114 28827360 PMC5594638

[B75] Zhao G. Winkler M. E. (1996). A novel alpha-ketoglutarate reductase activity of the *serA*-encoded 3-phosphoglycerate dehydrogenase of *Escherichia coli* K-12 and its possible implications for human 2-hydroxyglutaric aciduria. J. Bacteriol. 178, 232–239. 10.1128/JB.178.1.232-239.1996 8550422 PMC177644

[B76] Zhu L. Mack C. Wirtz A. Kranz A. Polen T. Baumgart M.. (2020). Regulation of γ-aminobutyrate (GABA) utilization in *Corynebacterium glutamicum* by the PucR-type transcriptional regulator GabR and by alternative nitrogen and carbon sources. Front. Microbiol. 11:544045. 10.3389/fmicb.2020.544045 33193127 PMC7652997

[B77] Zhu N. Xia H. Yang J. Zhao X. Chen T. (2014). Improved succinate production in *Corynebacterium glutamicum* by engineering glyoxylate pathway and succinate export system. Biotechnol. Lett. 36, 553–560. 10.1007/s10529-013-1376-2 24129953

